# Y chromosome polymorphisms may contribute to an increased risk of male-induced unexplained recurrent miscarriage

**DOI:** 10.1042/BSR20160528

**Published:** 2017-03-27

**Authors:** Yan Wang, Gang Li, Man-Zhen Zuo, Jun-Hua Fang, Hai-Rong Li, Dan-Dan Quan, Lu Huang, Ping-Ping Peng

**Affiliations:** 1 Department of Gynecology and Obstetrics, The People’s Hospital of Three Gorges University, The First People’s Hospital of Yichang, Yichang 443000, P. R. China; 2 Department of Neurosurgery, The People’s Hospital of Three Gorges University, The First People’s Hospital of Yichang, Yichang 443000, P. R. China; 3 Radiology Department, Traditional Chinese Medicine Hospital of Zhijiang, Yichang, 443000, P. R. China

**Keywords:** group D/G, inv(9), unexplained recurrent miscarriage, Yqh-, Yqh+, Y chromosome polymorphism, 1/9/16qh+

## Abstract

The present study aims to explore the relationship between the Y chromosome polymorphisms (1qh+, inv(9), 9qh+, 16qh+, group D/G, Yqh– and Yqh+) and the risk of unexplained recurrent miscarriage (URM). A total of 507 couples with URM were recruited as case group and 465 healthy couples as control group. The Y chromosome polymorphisms of the male individuals were analysed with the G-banding technique, and the results of the chromosome G-banding analysis were determined using the International Naming Standards of Human Genetics (ISCN). Logistic regression analysis was used to analyse the risk factors for URM. The detection rate of Y chromosome polymorphisms in the case group (12.03%) was higher than that in the control group (2.15%). Y chromosome polymorphisms were detected at significantly higher rates in the case group than in the control group. Using the normal Y chromosomes in individuals of the case group as reference, the partners of their counterparts were more likely to experience miscarriage. The couples who were Y chromosome-polymorphism carriers had shorter gestational age, increased frequency of URM and longer average interval between pregnancies. The results of logistic regression analysis revealed that Y chromosome polymorphisms, shorter gestational age, a higher frequency of miscarriage and longer pregnancy interval were independent risk factors for URM. Y chromosome polymorphisms may be associated with the risk of URM and may play an important role in the development of URM.

## Introduction

Recurrent miscarriage (RM) is defined as three or more consecutive miscarriages before 20 weeks of gestation with a foetus weighing <500 g [1,2]. However, it is pertinent to state here that the international standard of RM is the loss of two or more consecutive pregnancies [3]. For the past few years, the global incidence of RM in women of childbearing age has increased significantly, accounting for 2 to 5% of the total number of pregnancies [4]. Due to the complex mechanism and some other unexplained elements, the risk factors of RM still remain undefined [5,6]. It was reported that the risk factors of RM include luteal phase defect during pregnancy, uterine anomalies and some anatomic disorders of the uterine cavity, as well as possible factors such as thyroid dysfunction, chromosomal abnormalities and autoimmune disorders, among which chromosomal abnormalities are one of the common factors contributing to early RM [7,8]. Despite this, the risk factors for nearly 50% of RMs still remain a mystery; this type of RM is commonly known as unexplained RM (URM).

A chromosome polymorphism is defined as the variations in chromosome morphology, including changes in their size, shape and number, which can commonly occur [9,10]. Therefore, the Y chromosome, which is acrocentric and contains a large proportion of heterochromatin, is likely to exhibit morphological changes [11]. However, such structural changes often occur in one chromosome of a homologous pair, and Y chromosome polymorphism specifically refers to the increase or decrease or repeat in the chromosome group D/G coupled with short arm variations, variations in the fluorescence intensity of the centromere region of chromosomes 1, 9 and 16 or lengthening or shortening of the constriction area [12,13]. Spermatogenesis has been reported to be controlled by a network of genes located on the Y chromosome, and previous investigations have shown that certain chromosome number and structural abnormalities were closely related to male infertility [14,15]. With the number of infertile patients increasing, more and more focus has been put on the relationship between Y chromosome polymorphisms and URM for genetic counselling and reproductive research. Mostly, chromosomes differ in size and location of heterochromatin at the 1qh, 9qh and 16qh regions and classical euchromatic variants of 9q12/qh+ were suggested to be associated with RM [16]. In a Mexican study of 158 couples with URM, polymorphic variants in the constitutive heterochromatic regions at the 1qh+, 9qh+ and 16qh+ chromosomes were found in 25 couples (15.82%), at the Yqh+ chromosome in 21 couples (13.29%) and at group D/G in 12 couples (7.59%) [17]. A previous research revealed that in a total of 26 patients with poor semen status with chromosome polymorphism, there were six with polymorphisms at the Yqh+ chromosome, seven at the Yqh– chromosome, one at the 16qh+ chromosome and six at the inv(9) (p11q12) respectively [18]. These indicate that chromosome polymorphism might play a significant, yet unknown role in RM. Therefore, the present study was undertaken with the aim to analyse the association between Y chromosome polymorphisms (1qh+, inv(9), 9qh+, 16qh+, group D/G, Yqh– and Yqh+) and the carriers whose partner suffers from URM, in hope to provide a reference for studies on URM aetiology.

## Materials and methods

### Study subjects

A total of 1014 cases (507 couples) consisting of patients with URM and their respective spouses were recruited from the People’s Hospital of Three Gorges University (China), the First People’s Hospital of Yichang (China) and Department of Genetics and Fertility Centre (China) from August 2008 to December 2011 as the case group. All the included spouses of patients had a gestation time of <3 months, which was confirmed by ultrasound evaluation. The subjects in the case group were in the mean age of 29.3 ± 4.7 years and met the inclusion criteria as follows: (i) their spouses experienced three or more miscarriages [19]; (ii) no abnormal reproductive tract anatomy; (iii) normal endocrine function (female) and semen (male) testing results; (iv) the rest results of anti-TORCH virus series (Rubella virus, RuV; Cytomegalovirus, CMV; Herpes simplex virus, HSV), *Toxoplasma gondii*, anti-nuclear antigen, anti-cardiolipin antigen and anti-husband cytotoxicity were all negative [20]; (v) no reproductive tract or systemic inflammatory response; (vi) no thrombotic disease or tendency. The clinical characteristics of the women in the case group, including gestational age, frequency of miscarriage and pregnancy interval were recorded. Gestational length at the time of miscarriage was based on the crown-rump length (CRL) of the dead embryo by ultrasound. Interval between pregnancies was defined as the period of time between the first preterm birth and subsequent conception. Both gestational length at the time of miscarriage and interval between pregnancies were collected from medical records and patients’ questionnaires.

The control group included 930 cases (465 couples) of normal pregnant women and their respective partners, with an average age of 28.4 ± 3.3 years. These couples met the requirements of no miscarriage history and at least one child with no pregnancy-related complications. There was no significant difference in the baseline characteristics between the case group and the control group (all *P*>0.05). Patients were excluded from the present study if they had a history including drug use; severe cardiovascular disease; chronic pain; reproductive tract abnormalities; endocrine abnormalities or a family medical history; serious liver or kidney dysfunction; severe cardiopulmonary dysfunction; reoperation due to complications within 3 months after surgery; congenital abnormalities of gynaecological anatomy or cervical abnormalities; depression; neuropsychiatric dysfunction or other complications.

Additionally, all subjects were informed and signed an informed consent. The study was approved by the Ethics Committee of The People’s Hospital of Three Gorges University (China) and The First People’s Hospital of Yichang (China).

### Sample collection

First, blood lymphocyte cultures were prepared. Peripheral blood (0.8 ml) was extracted, placed in a heparin tube and then inoculated into a lymphocyte culture aseptically. The blood was injected as drops to avoid haemolysis. After gently mixing the cultures and incubating them at 37°C in an incubator for 72 h, two drops of 50 μg/ml colchicine were added with a 5-ml syringe to arrest the lymphocytes in metaphase 1 h before the end of the culture period.

For the chromosome specimen culture, cells were extracted after the cultures were centrifuged. The obtained peripheral blood lymphocytes were permeated for 20 min with hypotonic 0.075% mol/l potassium chloride (KCl) and were successively immobilized twice in a mixture of methanol and glacial acetic acid. With gentle pipetting, a cell suspension was ultimately obtained, and the appropriate cell suspension was dripped on a clean, wet slide (3–4 drops per sheet) to be dried under the outer-edge flame of alcohol lamp.

### G-banding analysis of chromosome karyotype

The prepared specimen was digested with trypsin (Nanjing KGI Biological Development Co., Ltd., Nanjing, China), which was pre-warmed at 37°C. Following the trypsin digestion, the specimen was rinsed gently with tap water and then stained with Giemsa (Beijing Ding Guo Chang Sheng Biotechnology, LLC, Beijing, China) for 3 to 5 min, rinsed and dried. Cells with intact morphology, well-dispersed chromosomes and clear bands in metaphase were randomly selected and viewed under a microscope (Olympus, Japan); karyotypes were enlarged and clipped. Samples with clear and different depth bands on the chromosome were desirable.

The standard required for karyotype analysis was the presence of 30 or more cells in metaphase in five pictures from each case available for analysis. Nearly, 20 metaphases were counted and 5–10 karyotypes were analysed for each sample. Chimaeric karyotype required double counting and analysis [21]. Results were determined by banding conditions based on the International Naming Standards of Human Genetics (ISCN, 2013) [22]. The interpretations were as follows: (i) unacceptable: blurred chromosome bands that can not be identified; (ii) acceptable: chromosomes 2, 4, 6, 13, 17, 20 and 22 were observed under 100× magnification in a light microscope and the chromosomes in areas with characteristic chromosome bands were not very clear but identifiable; (iii) best: chromosome bands with clear outlines, complete shapes, significant intermediate depths and no microhair, which were more conducive for karyotype analysis.

### Statistical analysis

Statistical package for the Social Sciences (SPSS) 19.0 (SPSS Inc., Chicago, IL, U.S.A.) was used for statistical analysis. Comparisons for count data presented as the ratio or rate between the two groups were made using χ^2^ test. Comparisons for measured data presented as the mean ± S.D. were made using a *t* test. A value of *P*<0.05 was considered statistically significant.

## Results

### Comparisons of baseline characteristics between the case and control groups

Among the 507 couples (aged between 22 and 36 years; mean age: 29.3 ± 4.7 years) in the case group, there were 136 couples with one or both sides of the couple suffering from obesity, 284 couples with one or both sides of the couple having a smoking history and 245 couples with one or both sides of the couple having a drinking history. Among the 465 couples (aged between 21 and 34 years, mean age: 28.4 ± 3.3 years) in the control group, there were 131 couples with one or both sides of the couple suffering from obesity, 218 couples with one or both sides of the couple having a smoking history and 221 couples with one or both sides of the couple having a drinking history. No statistical difference was found in the mean age, obesity, drinking history or smoking history between the cases in the two groups (*P*>0. 05) (Table 1).

**Table 1:** Comparisons of baseline characteristics between the case and control groups

Characteristic	Case group (*n*=507 couples)	Control group (*n*=465 couples)	*t*/χ2	*P*
Mean age (years)	29.3 ± 4.7	28.4 ± 3.3	1.142	0.254
Obesity (yes/no)	136/371	131/334	0.221	0.638
Smoking (yes/no)	248/259	218/247	0.402	0.526
Drinking (yes/no)	245/262	221/244	0.062	0.804

### Association between Y chromosome polymorphisms and URM risk

The detection rate of Y chromosome polymorphisms in the case group (12.0%, 61/507) was higher than that in the control group (2.2%, 10/465) (*P*<0.05). The detection rates of all Y chromosome polymorphisms (1qh+, inv(9), 9qh+, 16qh+, group D/G, Yqh– and Yqh+) were also higher in the case group than in the control group (all *P*<0.05). Compared with the male subjects with normal Y chromosomes in the case group, the partners of those men carrying Y chromosome polymorphisms of 1qh+ (1.38% compared with 0.22%, odds ratio (OR) (95% confidence interval (95%CI)) =6.496 (0.796–53.030), *P*=0.045), inv(9) (1.97% compared with 0.43%, OR (95%CI) =4.658 (1.015–21.380), *P*=0.030), 9qh+ (1.18% compared with 0.00%, OR (95%CI) =12.070 (0.677–214.900), *P*=0.019), 16qh+ (1.58% compared with 0.22%, OR (95%CI) =7.439 (0.926–59.740), *P*=0.027), group D/G (2.96% compared with 1.08%, OR (95%CI) =2.805 (1.011–7.781), *P*=0.039), Yqh– (1.78% compared with 0.22%, OR (95%CI) =3.386 (1.058–66.480), *P*=0.016), Yqh+ (1.18% compared with 0.00%, OR (95%CI) =12.070 (0.677–214.900), *P*=0.019) were all more likely to have a risk of miscarriage (all *P*<0.05) (Table 2).

**Table 2:** Distribution of Y chromosome polymorphisms between the case and control groups (*n* (%))

Y chromosome	Case group (*n*=507 couples)	Control group (*n*=465 couples)	χ^2^	*P*	OR	95%CI
Normal Y chromosome	446 (87.97%)	455 (97.85%)	Ref.	Ref.	Ref.	Ref.
1qh+	7 (1.38%)	1 (0.22)	4.040	0.045	6.496	0.796–53.030
inv(9)	10 (1.97%)	2 (0.43)	4.732	0.030	4.658	1.015–21.380
9qh+	6 (1.18%)	0 (0.00)	5.537	0.019	12.070	0.677–214.900
16qh+	8 (1.58%)	1 (0.22)	4.911	0.027	7.439	0.926–59.740
Group D/G	15 (2.96%)	5 (1.08)	4.269	0.039	2.805	1.011–7.781
Yqh–	9 (1.78%)	1 (0.22)	5.798	0.016	3.386	1.058–66.480
Yqh+	6 (1.18%)	0 (0.00)	5.537	0.019	12.070	0.677–214.900

### Association between Y chromosome polymorphisms and clinical characteristics of URM

**Figure 1:**
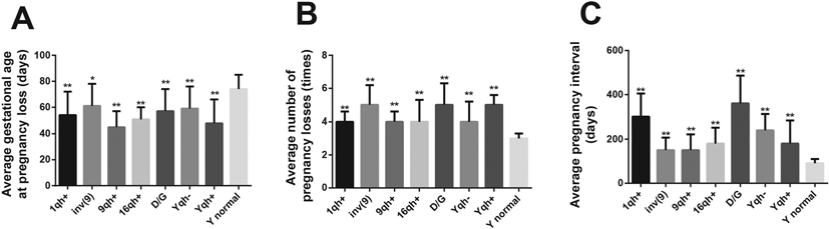
Association between Y chromosome polymorphisms and clinical features of patients with UMR (**A**) Association between Y chromosome polymorphisms and average gestational age; (**B**) Association between Y chromosome polymorphisms and average number of miscarriage; (**C**) Association between Y chromosome polymorphisms and average pregnancy interval; ^*^, compared with the normal Y chromosome, *P*<0.05; ^**^, compared with the normal Y chromosome, *P*<0.001.

When compared with the partners of male subjects with normal Y chromosomes, the partners of those men with Y chromosome polymorphisms of 1qh+, inv(9), 9qh+, 16qh+, group D/G, Yqh– and Yqh+ usually had shorter gestational age (74 ± 11 compared with 54 ± 18, 61 ± 17, 45 ± 12, 51 ± 9, 57 ± 17, 59 ± 17, 48 ± 18, all *P*<0.05), a higher frequency of miscarriage (3 ± 0.3 compared with 4 ± 0.6, 5 ± 1.2, 4 ± 0.6, 4 ± 1.3, 5 ± 1.3, 4 ± 1.2, 5 ± 0.6, all *P*<0.05) and longer interval between pregnancies (91 ± 19 compared with 300 ± 105, 150 ± 57, 150 ± 69, 180 ± 76, 360 ± 126, 240 ± 72, 176 ± 72, all *P*<0.05) (all *P*<0.05) (Table 3 and Figure 1).

**Table 3:** Association between Y chromosome polymorphisms and clinical characteristics of URM

Y chromosome	Case group *n* (%)	Average gestational age (days (S.D.))	Average number of miscarriages (*n* (S.D.))	Average pregnancy interval (days (S.D.))
Normal Y chromosome	446 (88.0%)	74 (11)	3 (0.3)	91 (19)
1qh+	7 (1.38)	54 ± 18^**^	4 ± 0.6^**^	300 ± 105^**^
inv(9)	10 (1.97)	61 ± 17^*^	5 ± 1.2^**^	150 ± 57^**^
9qh+	6 (1.18)	45 ± 12^**^	4 ± 0.6^**^	150 ± 69^**^
16qh+	8 (1.58)	51 ± 9^**^	4 ± 1.3^**^	180 ± 76^**^
Group D/G	15 (2.96)	57 ± 17^**^	5 ± 1.3^**^	360 ± 126^**^
Yqh–	9 (1.78)	59 ± 17^**^	4 ± 1.2^**^	240 ± 72^**^
Yqh+	6 (1.18)	48 ± 18^**^	5 ± 0.6^**^	176 ± 72^**^

*, compared with the normal Y chromosome, *P*<0.01; **, compared with the normal Y chromosome, *P*<0.001.

### Logistic regression analysis of risk factors for URM

Logistic regression analysis was used to analyse risk factors for URM. Average gestational age, average number of miscarriage, average pregnancy interval and Y chromosome polymorphisms (1qh+, inv(9), 9qh+, 16qh+, group D/G, Yqh– and Yqh+) were taken into logistic regression analysis. The results revealed that Y chromosome polymorphisms, shorter gestational age, a higher frequency of miscarriage and longer pregnancy interval were independent risk factors for URM (all *P*<0.05) (Table 4).

**Table 4:** Logistic regression analysis of risk factors for URM

Y chromosome	B	S.E.M.	Wald	Sig	Exp (B)	95%CI
Y chromosome polymorphism	2.339	1.112	4.419	0.036	10.366	1.171–91.740
Shorter gestational age	–0.035	0.01	11.426	0.001	0.966	0.947–0.986
Higher frequency of miscarriage	0.831	0.357	5.425	0.02	2.295	1.141–4.617
Longer pregnancy interval	0.024	0.006	18.167	<0.001	1.024	1.013–1.036

B, β; Sig, significance; Wald, regression coefficients.

## Discussion

The relationship between the Y chromosome polymorphisms (1qh+, inv(9), 9qh+, 16qh+, group D/G, Yqh–, Yqh+) and URM risk is investigated in the present study. The results demonstrated that the prevalence of Y chromosome polymorphism is higher in the group, in which the female partner suffered from URM. The Y chromosome polymorphisms are analysed with the G-banding technique, which established that there is a significantly higher detection rate of Y chromosome polymorphisms (1qh+, inv(9), 9qh+, 16qh+, group D/G, Yqh–, Yqh+) in the case group than in the control group. And by comparing baseline characteristics between the case and control groups, no significant difference was noted in the mean age, obesity, smoking history or drinking history. The Y chromosome polymorphisms are always heterochromatin variations, especially the highly repetitive DNA that is constitutively heterochromatin and the incidence is estimated to be 2.6% [11]. In recent years, research has demonstrated that Y chromosome polymorphisms could contribute to homologous chromosome pairing and chromosome segregation. However, Y chromosome polymorphisms could also discourage homologous chromosome pairing during the cell division phase, thus causing disorders such as cell division disorder, embryonic developmental disorder, teratogenic disorders, stillbirth and miscarriage [23,24]. The clinical symptoms of URM are further analysed and compared with the characteristics of individuals carrying normal Y chromosomes. The spouses of those carrying the Y chromosome polymorphisms (1qh+, inv(9), 9qh+, 16qh+, group D/G, Yqh–, Yqh+) exhibited a shorter gestational age, higher frequency of miscarriage and longer interval between pregnancies.

In the present study, the detection rate for the chromosome short arm trabant polymorphism for karyotype group D/G is 50% higher in the case group than in the control group. A higher number of D/G trabants in humans could lead to chromosome rearrangement, thus causing abnormal meiosis, an increased number of gametes and miscarriage [25]. Karyotypes (1qh+, 9qh+, 16qh+) are also significantly more prevalent in the case group in the study than in the control group. The increased heterochromatin length in these three karyotypes can lead to chromosome non-disjunction, foetal chromosomal abnormalities and subsequently, miscarriage [26]. It is reported that the karyotype inv(9) can cause miscarriage and stillbirth, and inv(9) carriers are likely to have structural abnormalities with unbalanced gametes. The prevalence of the karyotype Yqh+ is 1.18% in the case group, whereas it is not detected in the control group. The karyotype Yqh– is eight times more prevalent in the case group than the control group. A cytogenetic study shows that increase (Yqh+) or decrease (Yqh–) in the heterochromatin on the long arm of the Y chromosome can lead to mitotic errors, thus causing stillbirth or miscarriage [27]. The frequencies of Y chromosome polymorphism of 1qh+, 9qh+, 16qh+, group D/G and Yqh+ in the case group of our study (1.38%, 1.18%, 1.58%, 2.96%, 1.18% and 2.96 respectively) were lower than the previous data from a Mexican study of 158 couples with RM (6.96%, 5.70%, 3.16%, 7.61% and 12.29% respectively) [17]. A previous study revealed the cytogenetic effects on patients with infertility in a Turkish population, and the 1qh+, 16qh+, Yqh+ and inv(9) polymorphisms had frequencies of 0.5%, 1.5%, 1.82% and 0.5% respectively, different from the present study in URM patients (1.38%, 1.58%, 1.18% and 1.97% respectively) [16]. Though the data of these groups were not completely comparable, these findings revealed that Y chromosome polymorphism may play a pivotal role in URM. The results were confirmed by our logistic regression analysis that Y chromosome polymorphisms, shorter gestational age, higher frequency of miscarriage and longer pregnancy interval were independent risk factors for URM. As for other factors, we know from the previous data that the expression of protein kinase plays an implicated role in the pathogenesis of trisomic pregnancy [28]. Furthermore, *eNOS* gene polymorphism may regulate eNOS expression and was correlated with RM in an Indian population [29]. As for our study, the molecular mechanism of Y chromosome remains unknown and warrants further study.

In conclusion, our findings indicate that Y chromosome polymorphisms may be associated with the risk of URM, may act an important role in the development of URM. The detection of structural abnormalities in the Y chromosome may potentially serve as a predictor of the occurrence of miscarriage or foetal death among women. However, we failed to collect the time of first vaginal hemaorrhage and the time of uterine evacuation, which may affect the clinical application values of the present study. Finally, further research combining cytology, molecular genetics, genomics and other fields is urgently needed to clarify the mechanisms by which Y chromosome polymorphisms specifically relate to and lead to URM.

## Abbreviations

eNOS: endothelial nitric-oxide synthase
OR: odds ratio
RM: recurrent miscarriage
TORCH: Toxoplasma gondii, Rubella virus,Cytomegalovirus and Herpes simplex
URM: unexplained RM
95%CI: 95% confidence interval
